# Salvianolic Acid A Protects against Lipopolysaccharide-Induced Acute Lung Injury by Inhibiting Neutrophil NETosis

**DOI:** 10.1155/2022/7411824

**Published:** 2022-07-21

**Authors:** Qiang Liu, Cheng-long Zhu, Hui-ru Li, Jian Xie, Yu Guo, Peng Li, Zhen-zhen Zhao, Jia-feng Wang, Xiao-ming Deng

**Affiliations:** ^1^Jiangsu Province Key Laboratory of Anesthesiology, Xuzhou Medical University, Xuzhou, China; ^2^Faculty of Anesthesiology, Changhai Hospital, Naval Medical University, Shanghai, China; ^3^Faculty of Anesthesiology, Weifang Medical University, Weifang, Shandong Province, China

## Abstract

Salvianolic acid A (SAA) is one of bioactive polyphenol extracted from a Salvia miltiorrhiza (Danshen), which was widely used to treat cardiovascular disease in traditional Chinese medicine. SAA has been reported to be protective in cardiovascular disease and ischemia injury, with anti-inflammatory and antioxidative effect, but its role in acute lung injury (ALI) is still unknown. In this study, we sought to investigate the therapeutic effects of SAA in a murine model of lipopolysaccharide- (LPS-) induced ALI. The optimal dose of SAA was determined by comparing the attenuation of lung injury score after administration of SAA at three different doses (low, 5 mg/kg; medium, 10 mg/kg; and, high 15 mg/kg). Dexamethasone (DEX) was used as a positive control for SAA. Here, we showed that the therapeutic effect of SAA (10 mg/kg) against LPS-induced pathologic injury in the lungs was comparable to DEX. SAA and DEX attenuated the increased W/D ratio and the protein level, counts of total cells and neutrophils, and cytokine levels in the BALF of ALI mice similarly. The oxidative stress was also relieved by SAA and DEX according to the superoxide dismutase and malondialdehyde. NET level in the lungs was elevated in the injured lung while SAA and DEX reduced it significantly. LPS induced phosphorylation of Src, Raf, MEK, and ERK in the lungs, which was inhibited by SAA and DEX. NET level and phosphorylation level of Src/Raf/MEK/ERK pathway in the neutrophils from acute respiratory distress syndrome (ARDS) patients were also inhibited by SAA and DEX in vitro, but the YEEI peptide reversed the protective effect of SAA completely. The inhibition of NET release by SAA was also reversed by YEEI peptide in LPS-challenged neutrophils from healthy volunteers. Our data demonstrated that SAA ameliorated ALI via attenuating inflammation, oxidative stress, and neutrophil NETosis. The mechanism of such protective effect might involve the inhibition of Src activation.

## 1. Introduction

Acute lung injury (ALI)/acute respiratory distress syndrome (ARDS) is life-threatening acute diffuse inflammatory injury in lungs characterized by increased pulmonary vascular permeability, inflammatory cell infiltration, pulmonary edema, diffuse alveolar damage, and decreased respiratory volume [1, 2]. As a common cause of respiratory failure in critically ill patients, ALI/ARDS has different specific etiologies, including sepsis, gastric inhalation, trauma, and pneumonia, and is seen in approximately 10% of patients in intensive care units (ICUs) worldwide [3–5]. Pathological changes of ALI/ARDS involve diffuse alveolar damage along with accumulation of neutrophil infiltration in the bronchoalveolar space, alveolar hemorrhage, fibrin deposition, and collapse in some alveoli. In addition, alteration in endothelial and epithelial cells such as the destroyed alveolar epithelium and exfoliated basement membrane is also involved in typical histopathology [6–8]. Despite the apparent improvements in medical care drive from decades of continuous efforts to treat ALI/ARDS, mortality rates are still as high as 30-40% in most studies, owing to the lack of specific and effective therapies for the disease [9]. Thus, an urgent demand of effective therapies is required for ALI/ARDS.

As vital components of effector and regulatory circuits in the innate and acquired immune systems, neutrophils are the front line for recognizing and killing invading pathogens through degranulation, respiratory burst with superoxide production, enabling neutrophil elastase (NE) release and formation of neutrophil extracellular traps (NET) [10–12]. Neutrophil infiltration is an emblematic hallmark of ARDS in inflammatory lungs; although, activation of neutrophils is critical for host defense, and overzealous activation triggers more NETs, leading to lung damage [6, 13]. The release of NET from neutrophils is called as a multistep named NETosis. NETs are mainly constituted by cellular-free DNA, histones, and globular proteins such as myeloperoxidase (MPO), induced by viruses, ROS, coinfecting microorganisms, cytokines and chemokines, cationic antimicrobial peptides, and platelets in the infected and inflamed tissues [14, 15]. NETs immobilize pathogens, confine their metastasis, and facilitate antimicrobial defense via antimicrobial proteins [16]. However, excessive NETs conduce to the pathogenesis of numerous diseases, such as direct cell damage and delay in tissue repair, and involve infection, sepsis, autoimmunity, and diabetes, due to either excessive formation and/or impaired removal [17].

The Src family kinases (SFKs) are a group of intracellular proteins with tyrosine protein kinase activity, which plays a significant role in transducing extracellular signals into cell and accommodating diverse neutrophil functions, such as recruitment towards inflamed regions, adhesion to endothelium, NET formation, and ROS production [18–21]. On the other hand, inhibiting the phosphorylation and activity of SFKs and NET formation has been demonstrated to contribute to attenuated pulmonary inflammation in ARDS [22, 23].

**Figure 1:**
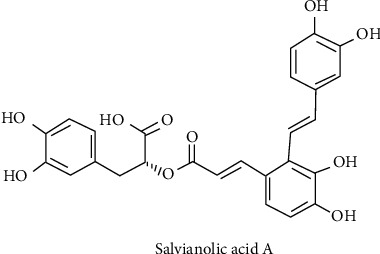
Structural formula of salvianolic acid A.

Xuebijing injection is a traditional Chinese medicine, mainly composed of five edible herb medicines, including Carthamus tinctorius, Paeonia veitchii, Ligusticum chuanxiong Hort, Salvia miltiorrhiza, and Angelica sinensis [24], which is widely used in the treatment of ARDS, sepsis ,and COVID-19, leading to a statistically significant improvement in clinical outcomes [25, 26]. Salvianolic acid A (SAA) (Figure 1), one major bioactive component of Salvia miltiorrhiza in Xuebijing Injection [27], possesses a variety of pharmacological effects, such as anti-inflammatory, antioxidative, prevents endothelial injury, and attenuates the production of ROS by inhibiting the Src signaling pathway [28, 29], as well as restraining cancer cell invasion [30]. These recent researches suggest that SAA might be a potential Src modulator. Yet, the impact of SAA on lung injury induced by LPS remains poorly understood. Therefore, our present study is performed to investigate whether SAA could eliminate the ALI induced by LPS in mice, a model widely used for ALI research [31–33], and explore the potential mechanisms associated with the protective effect of SAA against lung injury.

## 2. Materials and Methods

### 2.1. Animals and Patients

C57BL/6J mice (male, 8-10 weeks old) were obtained from the GemPharmatech Experimental Animal Corporation (Nanjing, China). All animals were maintained in a specific-pathogen-free laboratory animal environment and light dark cycle of 12 h. And they had free access to food and water freely. The protocol of the animal study was approved by the ethics committee on Biomedicine Research of Changhai Hospital, Shanghai, China, and conformed to the relevant rules and regulations.

Patients meeting clinical diagnostic criteria for ARDS were collected from the Intensive Care Unit of Changhai Hospital. Healthy volunteers aged 20–30 years who had not taken any medication within the previous 2 weeks were also recruited as healthy control. The experimental protocols are supported by the ethics committee on Biomedicine Research of Changhai Hospital.

### 2.2. *In Vivo* Experiments

The ALI model was established with lipopolysaccharide [34]. In brief, mice were anesthetized with sevoflurane (Hengrui, Lianyungang, Jiangsu, China). After cutting through the skin of the neck and then exposing the trachea, a trimmed sterile 31-gauge needle was inserted into the tracheal cavity. LPS (Sigma, St Louis, MO, USA) diluted in endotoxin-free saline was intratracheally (IT) injected at a dose of 10 mg/kg in 50 *μ*l saline.

The incision was then sewn up carefully. The sham group was treated with saline of the same volume. After 24 hours, the mice were sacrificed by cervical dislocation, and their lungs and bronchoalveolar lavage fluids (BALF) were collected.

### 2.3. Drug Administration

Endotoxin-free SAA (C26H22O10, CAS, 96574–01-05, purity ≥ 98% by HPLC) was purchased from Zheyan Biotechnology (Shanghai, China) and dissolved in sterile phosphate buffered saline (PBS). SAA or PBS was immediately administered to the mice by tail intravenous injection at the same volume after surgery. As a well-accepted drug in treating lung injury with strong clinical evidence, dexamethasone (DEX) was used as a positive control [35, 36]. In order to investigate the optimal therapeutic dose of SAA in alleviating acute lung injury in mice, 30 mice were randomly divided into the flowing five groups (*n* = 6 for each group): sham + PBS group, sham-operated mice were treated with PBS; LPS + PBS group (50 *μ*l), LPS mice were treated with PBS; and LPS + SAA group, LPS mice were treated with low, medium, or high doses of SAA (5, 10, or 15 mg/kg in 50 *μ*l). These doses of SAA were determined according to previous study [37]. Histopathologic tests in the lungs were compared to determine the optimal dose of SAA.

An optimal dose SAA was selected to further investigation. A total of 30 mice were randomly divided into 5 groups (*n* = 6 for each group): sham + PBS group, sham-operated mice were treated with PBS; sham + SAA group, sham-operated mice were treated with optimal dose SAA in 50 *μ*l; LPS + PBS group, LPS mice were treated with PBS (50 *μ*l); LPS + SAA group, LPS mice were treated with optimal dose SAA in 50 *μ*l; and LPS + DEX group, LPS mice were treated with DEX (5 mg/kg in 50 *μ*l).

### 2.4. *In Vitro* Experiments

Peripheral venous blood samples were collected from ARDS patients and healthy individuals, and the neutrophils were purified from peripheral blood using normative protocol by density gradient centrifugation with Ficoll-Hypaque (GE Healthcare, Little Chalfont, UK) and dextran as previously described [38, 39]. The PMN pellet was resuspended in DMEM supplemented with 10% FBS, 1% glutamine, and 1% penicillin/streptomycin solution at a concentration of 1 × 10^6^ cells/ml. Cells were incubated in polypropylene tubes to prevent adherence. Normal healthy neutrophils were stimulated with LPS (1 *μ*g/ml) (Sigma, St Louis, MO, USA) to simulate inflammatory changes and then treated with SAA (50 *μ*M) or DEX (1 *μ*M). ARDS neutrophils were treated with SAA (50 *μ*M) or DEX (1 *μ*M). YEEI peptide (Santa Cruz Biotechnology, sc-3052, 10 *μ*M), a synthetic agonist for Src family members, was also used to observe the role of Src in the effect of SAA. Twenty-one hours later, cells and supernatant were harvested after centrifugation.

### 2.5. Lung Histopathological Test

Lungs were fixed in 4% paraformaldehyde solution and subsequently processed into paraffin-embedded blocks and sectioned. Sections were stained with hematoxylin and eosin (HE) after deparaffinization and observed under light microscopy. A histopathological semiquantitative score was independently conducted and assessed by two pathologists according to the criteria based on the alveolar congestion, hemorrhage, the existence of assembling with inflammatory cells, and the thickness of the alveolar walls [40, 41]. Briefly, alveolar wall thickness, cellular infiltration, and hemorrhage were each scored from 0 (no injury) to 4 (maximal injury). The counts of each score were summed, and the results were recorded as the ALI score.

### 2.6. Enzyme-Linked Immunosorbent Assays

According to the manufacturer's instructions, cytokines levels of TNF-*α*, IL-6, and IL-1*β* in the BALF, as well as MPO-DNA complexes in supernatant of neutrophils were detected using the Invitrogen ELISA kits (Carlsbad, CA, USA).

### 2.7. Protein Concentration Determination

Collected by intratracheal injection with cold PBS, the BALF was centrifuged, and the protein concentration in the supernatant was assayed by utilizing the BCA detection kit (Thermo Scientific, Rockford, IL, USA). The absorbance was measured at 540 nm according to the operation instructions of the kit.

### 2.8. Immunofluorescence Detection

Immunofluorescence microscopy was handled to observe the NET formation and activated neutrophils in lung tissues. After retrieving antigen, the blocked sections with 10% donkey serum were incubated overnight with primary antibodies against MPO and histone H3 (1 : 100, Abcam, Cambridge, UK) at 4°C. Then, these sections were washed with PBS and treated with the secondary antibody (1 : 1000) for 1.5 h at room temperature. Ultimately, the sections were observed under fluorescence microscopy (Leica, Wetzlar, Germany).

### 2.9. Lung Wet-to-Dry Weight Ratio

The wet weight of lung tissues was determined immediately after sampling. Then, the lungs were incubated in an oven at 80°C to keep dry until the dry weight of the lung is in a constant weight state (the difference between the two weights should not exceed a certain allowable error). The pulmonary edema was evaluated by calculating the wet-to-dry ratio.

### 2.10. Flow Cytometry Detection

The cells in the BALF were stained with the anti-Ly6G-PE and anti-CD11b-APC antibodies (eBiosciences, San Diego, CA, USA) for cell calculation by flow cytometry operated on FACSCanto II flow cytometer (BD Bioscience, San Jose, CA, USA).

### 2.11. Western Blot

Lung tissues or neutrophils were lysed with RIPA buffer containing protease inhibitor and phosphatase inhibitor for protein collection. Then, the protein concentration was quantified using BCA Protein Quantitative Analysis Kit (ThermoScientific, Rockford, IL, USA). Appropriate amount of protein samples was separated by 10% SDS-polyacrylamide gel electrophoresis and transferred to PVDF (polyvinylidene fluoride) membrane. Membranes were blocked with 5% skimmed milk for 1 h and incubated overnight at 4°C with primary antibodies: Src, P-Src, c-Raf, P-c-Raf, MEK, P-MEK, ERK, and P-ERK (Cell Signal Technology, Beverly, MA, USA, diluted at 1 : 1000). After washing with TBST buffer, membranes were incubated with goat anti-mouse IgG-HRP and goat anti-rabbit IgG-HRP (Engibody Biotechnology, Milwaukee, WI, USA, diluted at 1 : 2000), for 2 h at room temperature. The blots were imaged by electrochemiluminescence (ECL) detection kit (Pierce, Rockford, IL, USA) and Imaging System (Bio-Rad, USA).

### 2.12. Oxidative Stress Assessment

The content of superoxide dismutase (SOD) and malondialdehyde (MDA) reflected the level of oxidative stress through the assay kits (JianCheng Bioengineering Institute, Nanjing, Jiangsu, China). In brief, an appropriate amount of lung tissue was mixed with the homogenate medium and centrifuged for 10 min at 3000-4000 r, and the supernatant was prepared into for direct detection at 532 nm.

### 2.13. Statistical Analysis

Statistical analysis was performed using the GraphPad Prism 8.3 (GraphPad Software Inc., CA, USA). The data was presented as mean ± standard deviation (SD) and compared using one-way analysis of variance (ANOVA). Then, Tukey's multiple comparison test was used for post hoc comparisons. Values of *p* < 0.05 were considered as statistically significant.

## 3. Results

### 3.1. The Optimal Dose of SAA in Treating Acute Lung Injury Induced by LPS

Lung tissues were harvested at 24 h after LPS injection for histopathologic assessment to screen the optimal SAA dose. As shown in (S Figure 1), in the LPS group, alveolar structure was severely damaged, along with enlarged lung septum, and increased inflammatory cells in lung stroma and alveoli. Compared with the LPS group, the damage of the lung tissues was significantly attenuated in all of the three SAA groups. The semiquantitative analysis showed that the lung injury scores in the 10 mg/kg and 15 mg/kg groups were similar group but were significantly lower than in the low dose group (5 mg/kg). Therefore, medium dose of SAA (10 mg/kg) was selected for further investigation as the optimal dose for SAA.

### 3.2. Salvianolic Acid A Ameliorated the Pathological Changes and Lung Injury Score Induced by LPS

**Figure 2:**
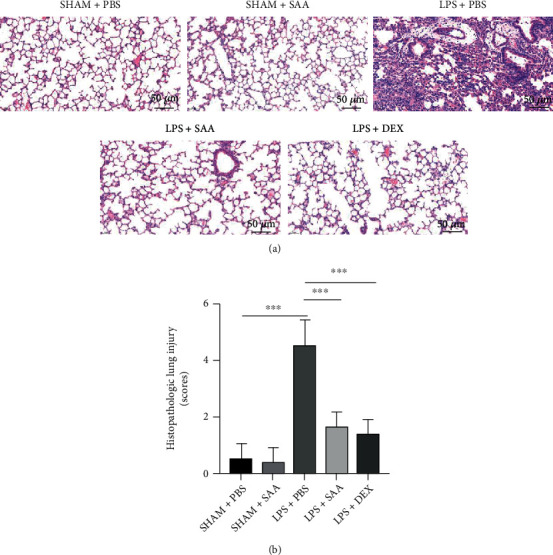
Effects of SAA at 10 mg/kg on LPS-induced histopathologic changes in the lungs. (a) Representative HE staining in histological sections of the lung at 24 h after ALI modeling established by LPS (10 mg/kg) and administration of SAA (10 mg/kg) or DEX (5 mg/kg) (Scale bar, 50 *μ*m). (b) The semiquantitative scores of the histopathologic changes (mean ± SD, ^∗∗∗^*p* < 0.001, *n* = 6, each group).

HE staining of damaged lung tissues was used to investigate the protective effect of SAA at 10 mg/kg. In order to exclude the drug-induced lung injury, a sham + SAA group was appended. The results showed that SAA has no harmful effect in lung function. Similarly, SAA was protective against LPS-induced acute lung injury as illustrated by the significant reduction in the destruction of alveolar structure and increasing in the infiltration of inflammatory cells (Figure 2(a)), when compared with the LPS group. The semiquantitative score of lung tissue pathological injury suggested that both of SAA and DEX could alleviate LPS-induced lung injury (*p* < 0.05) (Figure 2(b)).

### 3.3. Salvianolic Acid A Alleviated Pulmonary Edema and Leukocyte Infiltration in Mice with ALI

**Figure 3:**
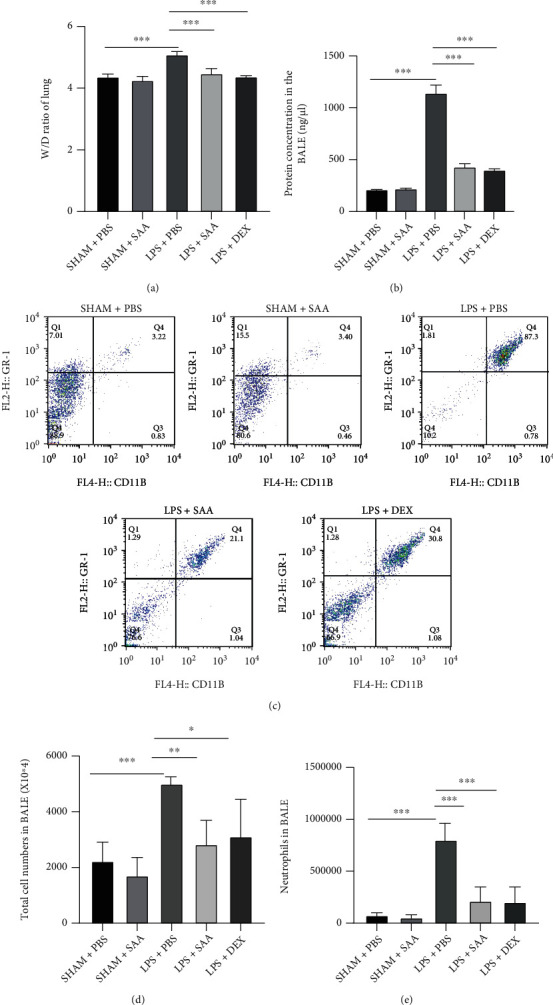
Effects of SAA on the lung edema and leukocyte infiltration in LPS-induced ALI mice. (a) Lung tissues were weighed to calculate the W/D ratio. (b) The total protein concentration in BALF was quantified via BCA assay at wavelength 540 nm. (c) The neutrophils proportion was detected by flow cytometry in BALF. (d) The total cells count was assayed by flow cytometry in BALF. (e) The neutrophils count was measured by flow cytometry in BALF (mean ± SD, ^∗^*p* < 0.05, ^∗∗^*p* < 0.01, ^∗∗∗^*p* < 0.001, *n* = 4 or 6 for each group).

Endothelial barrier function is disrupted during ALI, resulting in the infiltration of large amounts of fluid, macromolecules, and leukocyte into the lung mesenchyme. As shown in Figures 3(a) and 3(b), LPS challenge increased the wet-to-dry ratio of lung tissue and total protein concentration in BALF, while SAA and DEX reversed these changes induced by LPS. LPS also increased the number of total cells and neutrophils in BALF, while both SAA and DEX inhibited the infiltration of cells into BALF (Figures 3(c)–3(e)).

### 3.4. Salvianolic Acid A Suppressed LPS-Induced Inflammatory Responses and Oxidative Stress in the Lungs

**Figure 4:**
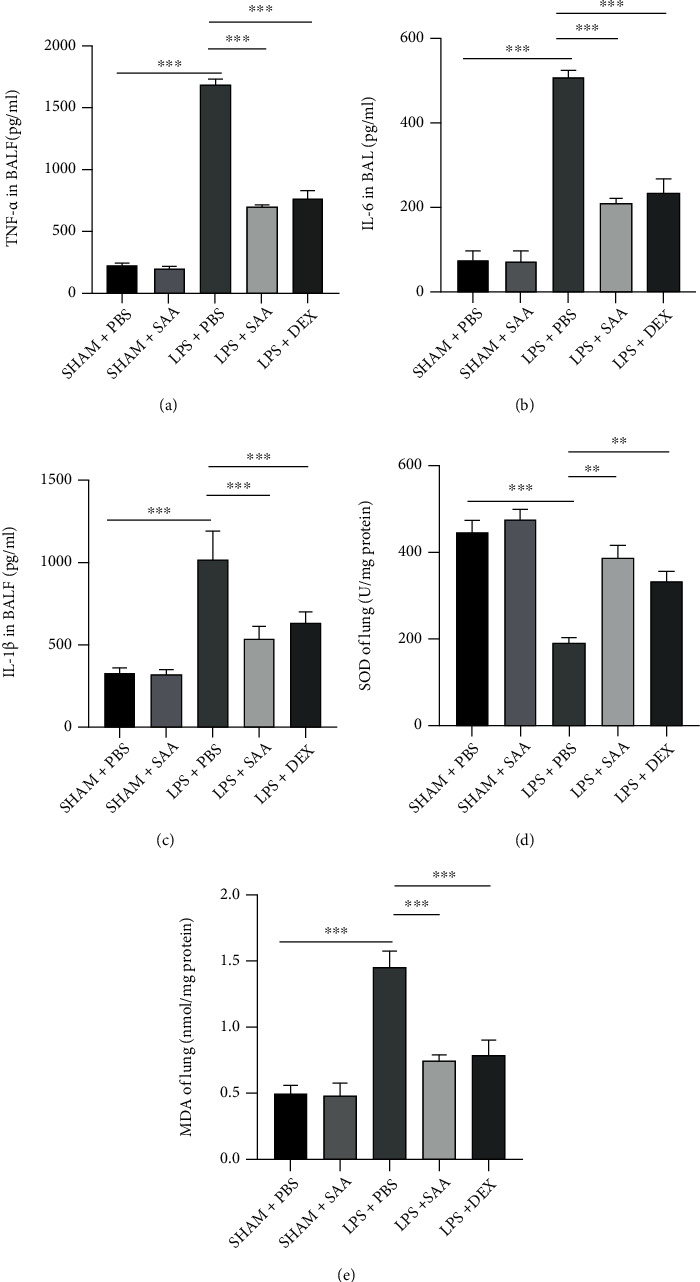
Effects of SAA on the production of inflammatory cytokines and oxidative stress in LPS-induced ALI mice. (a) TNF-*α* in BALF after ALI 24 h. (b) IL-6 in BALF after ALI 24 h. (c) IL-1*β* in BALF after ALI 24 h. (d) The activity of SOD in lung tissues challenged with LPS and treated with SAA or DEX. (e) MDA content in lung tissues challenged with LPS and treated with SAA or DEX. The values presented are mean ± SD (^∗∗^*p* < 0.01, ^∗∗∗^*p* < 0.001, *n* = 6 for each group).

LPS challenge induces excessive inflammation activation and oxidative responses in the lung tissue. ELISA assays demonstrated that inflammatory cytokines including TNF-*α*, IL-6, and IL-1*β* were remarkably increased at 24 h after LPS injection. SAA and DEX reduced these proinflammatory cytokines significantly to a similar extent (Figures 4(a)–4(c)). Oxidative stress in the lungs is measured by detecting the activity of SOD and the level of MDA, a product of lipid peroxidation via reactive oxygen species (ROS). LPS increased MDA level and decreased SOD activity, while SAA significantly increased SOD activity and decreased MDA level (Figures 4(d) and 4(e)).

### 3.5. SAA Attenuated NET Formation in LPS-Challenged Lungs

**Figure 5:**
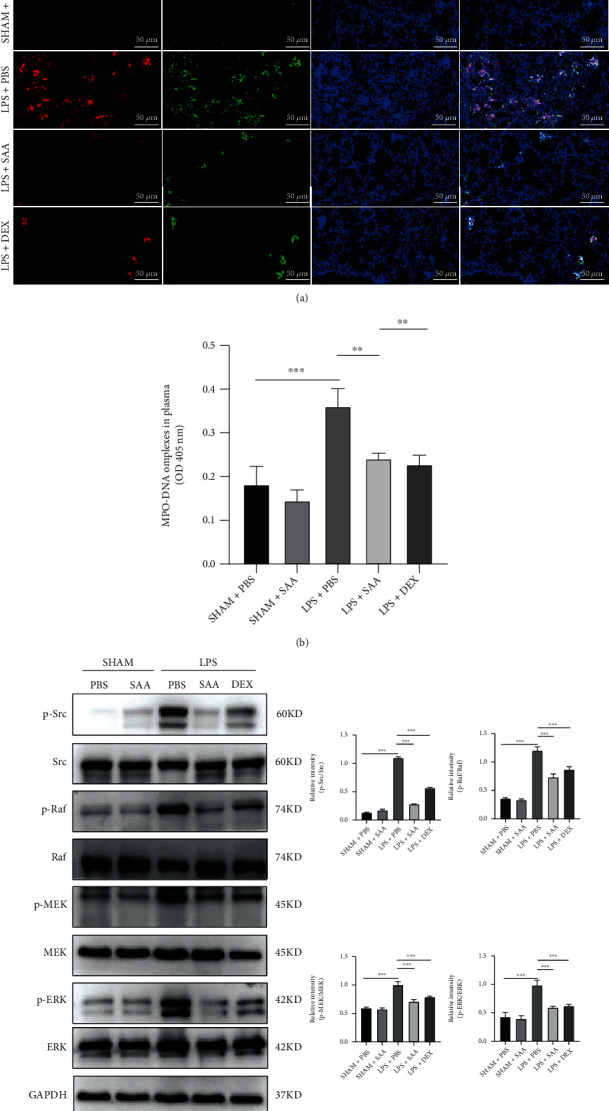
Effects of SAA on NETosis and Src/Raf/MEK/ERK signaling pathway in lung tissues and plasma of ALI mice. (a) Immunofluorescence assay of MPO and citrullinated histones (Cit-H3) in lung tissue at 24 h after ALI modeling and administration of SAA (10 mg/kg) or DEX (5 mg/kg) (scale bar, 50 *μ*m). (b) The levels of MPO-DNA complexes in the plasma of mice. (c) Protein expression in lung homogenate and the relative quantification of p-Src/Src, p-Raf/Raf, p-MEK/MEK, and p-ERK/ERK (mean ± SD, ^∗∗^*p* < 0.01, ^∗∗∗^*p* < 0.001, *n* = 4 or 6 for each group).

The pathological process of LPS-induced acute lung injury is accompanied by the formation of NET, which might be associated with oxidative stress. In order to elucidate the effect of SAA on the NETosis, we used immunofluorescence to measure the overlap of MPO and histone in lung tissues, and the result showed that SAA decreased NET formation of lung tissues (Figure 5(a)). Similarly, SAA and DEX decreased the NET level significantly in the plasma of ALI mice (Figure 5(b)). The activation of the Src kinase and the following signaling pathway plays a pivotal role in the production of ROS and NETs. Western blot showed that phosphorylation levels of Src, Raf, MEK, and ERK were all enhanced by LPS while SAA and DEX attenuated these activation (Figure 5(c)).

### 3.6. SAA Inhibited NET Formation and the Activation of Src Kinase in ARDS Neutrophils

**Figure 6:**
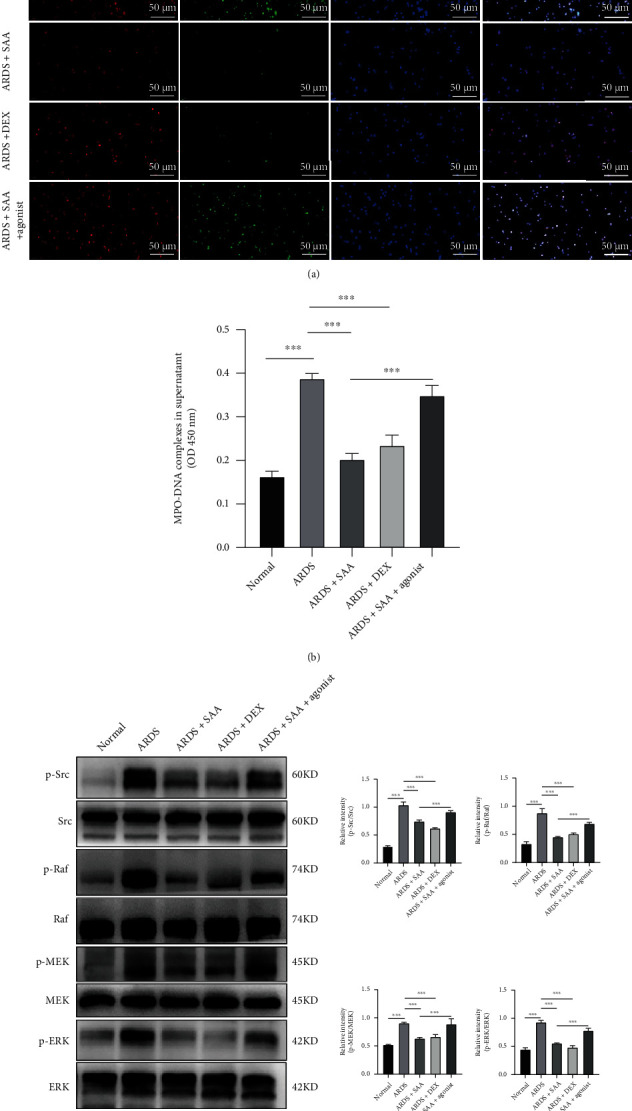
Effects of SAA on NETosis and Src/Raf/MEK/ERK signaling pathway in ARDS neutrophils. (a) Immunofluorescence assay of MPO and Cit-H3 in ARDS neutrophils at 21 h after treated with SAA (50 *μ*M) or DEX (1 *μ*M) and stimulated with Src family agonist (10 *μ*M) or not (scale bar, 50 *μ*m). (b) The levels of MPO-DNA complexes in the ARDS cells supernatant. (c) Protein expression in neutrophils lysate and the relative quantification of p-Src/Src, p-Raf/Raf, p-MEK/MEK, and p-ERK/ERK (mean ± SD, ^∗∗∗^*p* < 0.001; *n* = 3 for normal volunteers and 5 for ARDS patients).

In order to further confirm the effect of SAA on NET formation, we recruited 5 ARDS patients from ICU in our hospital and 3 additional healthy volunteers (mean aged 52 ± 2.94 years old, 2 males and 1 female) and isolated their neutrophils from the peripheral venous blood. There were 3 male and 2 female patients, with an average age of 52 ± 3.22 years old and mean PaO_2_/FiO_2_ of 155.6 ± 9.56 mm Hg. The cause of ARDS in 5 patients was all pulmonary infection (Table S1). The immunofluorescence assays showed that NETs were abundant in ARDS neutrophils at 21 h after culture *in vitro*, while SAA and DEX decreased the NET level significantly (Figures 6(a) and 6(b)). Interestingly, YEEI peptide almost completely reversed the protective effect of SAA. Similarly, SAA and DEX inhibited the phosphorylation level of Src, Raf, MEK, and ERK in ARDS neutrophils. Again, the Src agonist reversed the inhibitory effect of SAA (Figure 6(c)).

### 3.7. SAA Reduced NET Formation in LPS-Stimulated Healthy Neutrophils

**Figure 7:**
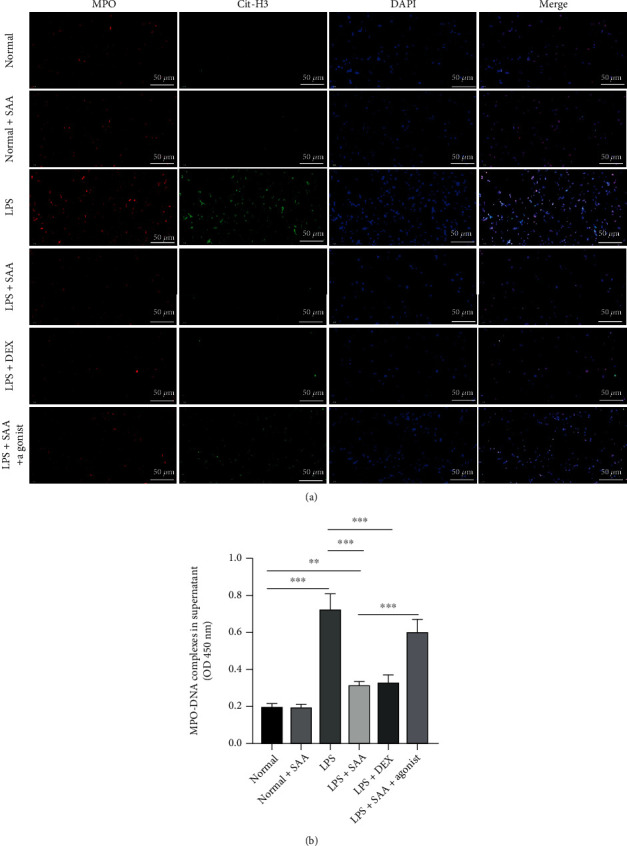
Effects of SAA on NETosis in normal healthy individual neutrophils. (a) Immunofluorescence assay of MPO and Cit-H3 in normal healthy individual neutrophils at 21 h after challenged with LPS (1 *μ*g/ml) and subsequent treatment. In the SAA treatment group, interference was performed or not with agonist (scale bar, 50 *μ*m). (b) The levels of MPO-DNA complexes in the normal healthy individual cells supernatant (mean ± SD, ^∗∗^*p* < 0.01; ^∗∗∗^*p* < 0.001; *n* = 6 for each group).

A further in vitro experiment was performed to investigate the effect of SAA on NET formation in healthy neutrophils stimulated with LPS. LPS stimulation increased the NET level in LPS-stimulated healthy neutrophils. SAA decreased NET formation similarly to DEX, while YEEI peptide reversed this protective effect (Figures 7(a) and 7(b)).

## 4. Discussion

Our current study demonstrated that SAA was protective against LPS-induced ALI. Specifically, SAA reduced the inflammatory response and inhibited oxidative stress in lungs of mice with LPS administration intratracheally. NETosis process and activation of the Src kinase were also inhibited by SAA in activated neutrophils from ARDS patients or LPS-stimulated healthy human neutrophils. Furthermore, Src kinase protein agonist reversed the protective effect of SAA against NETosis.

As we know, Chinese herbal medicine usually contains several various herbal components, but it is difficult to determine the specific effect of single component, which is important for pharmaceutical development. SAA is one of bioactive polyphenol extracted from a traditional Chinese herbal medicine Salvia miltiorrhiza, which is one of five main components of Xuebijing injection [42, 43]. It has been reported that Salvia miltiorrhiza played a protective role against diabetes, cancer, cardiovascular disease, nervous system disease, and inflammatory diseases with an anti-inflammatory, antioxidative, and antiapoptotic effects [44–47]. SAA has also been reported to attenuate injury of kidney disease, which can be attributed to anti-inflammatory and anti-xidative activities by inhibiting the NF-*κ*B and p38 MAPK signaling pathways and activating the Akt/GSK-3*β*/Nrf2 signaling pathway [37, 48]. SAA could also alleviate pulmonary arterial remodeling in pulmonary artery endothelial cells [49]. ROS production and angiotensin II-induced proliferation of HUVECs might by inhibited by attenuating Src phosphorylation [29]. Additionally, SAA could inhibit the invasion and migration of oral squamous cell carcinoma by inhibiting the c-Raf/MEK/ERK pathways [50]. In the present study, SAA was found to inhibit inflammation and oxidation in LPS-induced lung injury with a similar effect to DEX and provided new evidence for ARDS therapy via traditional herb. In addition, the analog salvianolic acid B also has similar activity. Zhao et al. demonstrate that salvianolic acid B (SAB) attenuates LPS-induced ALI through inhibition of apoptosis, oxidative stress, and inflammation in rats and therefore exerts protective effect against ALI, but they fail to reveal the possible mechanism [51]. Yang et al. suggest that through decreasing the expressions of the channel kinase TRPM6 and TRPM7, SAB protects against ALI in septic rats [52]. The effect of SAB on ALI has been reported, but the effect of SAA on ALI has not been reported; so, this study is a complement to the fact that Salvia miltiorrhiza can treat ALI.

Neutrophils may defend against pathogen insults via phagocytosis and the acceleration of ROS, proteases, and NETosis [53]. When the neutrophils are stimulated, activation of the PKC and c-Raf/MEK/ERK signaling pathway promotes production of ROS, closely required in NET information [54]. The protein-arginine deiminase 4 (PAD4) is subsequently activated, and citrullinate arginine on histones induces chromatin decondensation. Then, cytoplasmic azurophilic granules release MPO and NE, which promote chromatin depolymerization and leading to the release of NET eventually [55], while NET has a positive effect on host defense, excessive formation and delayed clear may induce organ injury and are detrimental to the host [56]. Some scholars found that the signaling pathway of NET formation after *β*-glucan particle internalization involves Src family kinase/Syk and ROS [57]. In addition, inhibition of phosphorylation of Src family kinases helps reduce NET release and exacerbation of lung injury in mice [22]. Our results showed that SAA might inhibit LPS-induced NET formation and protect against LPS-induced lung injury. Inhibitory effect of NET formation and Src/Raf/MEK/ERK pathway activation by SAA was also observed in LPS-challenged neutrophils and neutrophils isolated from ARDS patients. Furthermore, in vitro study demonstrated that such protective effect of SAA was reversed by Src agonist; thus, inhibition of Src pathway might be involved in the mechanism of SAA protection against NET formation. The mechanism of SAA regulation is related to protein-protein interactions mediated by the SH2 structural domains of the Src family kinases Src and Lck [58]. Our studies suggest that SAA treatment inhibits NET formation by suppressing the Src signaling pathway in vivo and in vitro. However, the specific mechanism of the protective effect of SAA on acute lung injury via Src signaling has not been elucidated.

Moreover, SAB was reported that it mainly interferes with MPO activity to relieve NET formation, and Salvia miltiorrhiza may preferentially interfere with NET in the early stage of NET formation [59]. However, tanshinone has been reported to increase ROS production in macrophages [60]. Recently, James et al. found that Src kinase inhibition with dasatinib impairs neutrophil function and clearance of Escherichia coli infection in a murine model of acute lung injury [61]. Neutrophil activation is usually considered as a double-edged sword in infective diseases. Inhibition of Src kinase may compromise the bactericidal capacity of neutrophils, but in LPS-induced ALI, the inhibition of Src kinase and NET formation may attenuated lung injury. Similar effect of Src blockade or NET inhibition in acute lung injury has also been reported by others [22, 62].

## 5. Conclusions

In conclusion, our study demonstrated that SAA, a nature component extracted from Salvia miltiorrhiza, was protective against LPS-induced ALI and NETosis in mice. The mechanism might involve the inhibition of Src activation. However, as a multifunctional herbal drug, considering the diversity of molecular mechanisms, the molecular target of SAA remains to be further investigated.

## Data Availability

The datasets during and/or analyzed during the current study are available from the corresponding authors on reasonable request.

## Supplementary Materials

Supplementary Materials Supplementary Figure 1: effects of SAA at different doses on LPS-induced histopathologic changes in the lungs. Supplementary Table S1: information of patients with acute respiratory distress syndrome.
