# Supplementary material for: Salvianolic Acid A Protects against Lipopolysaccharide-Induced Acute Lung Injury by Inhibiting Neutrophil NETosis
Source: Oxid Med Cell Longev. 2022 Jul 21;2022:7411824. doi: 10.1155/2022/7411824 (PMC9334034; doi:10.1155/2022/7411824)
Supplement: Supplementary Materials — Supplementary Figure 1: effects of SAA at different doses on LPS-induced histopathologic changes in the lungs. Supplementary Table S1: information of patients with acute respiratory distress syndrome. [file 7411824.f1.docx]

**Supplementary files**

**Figure S1**


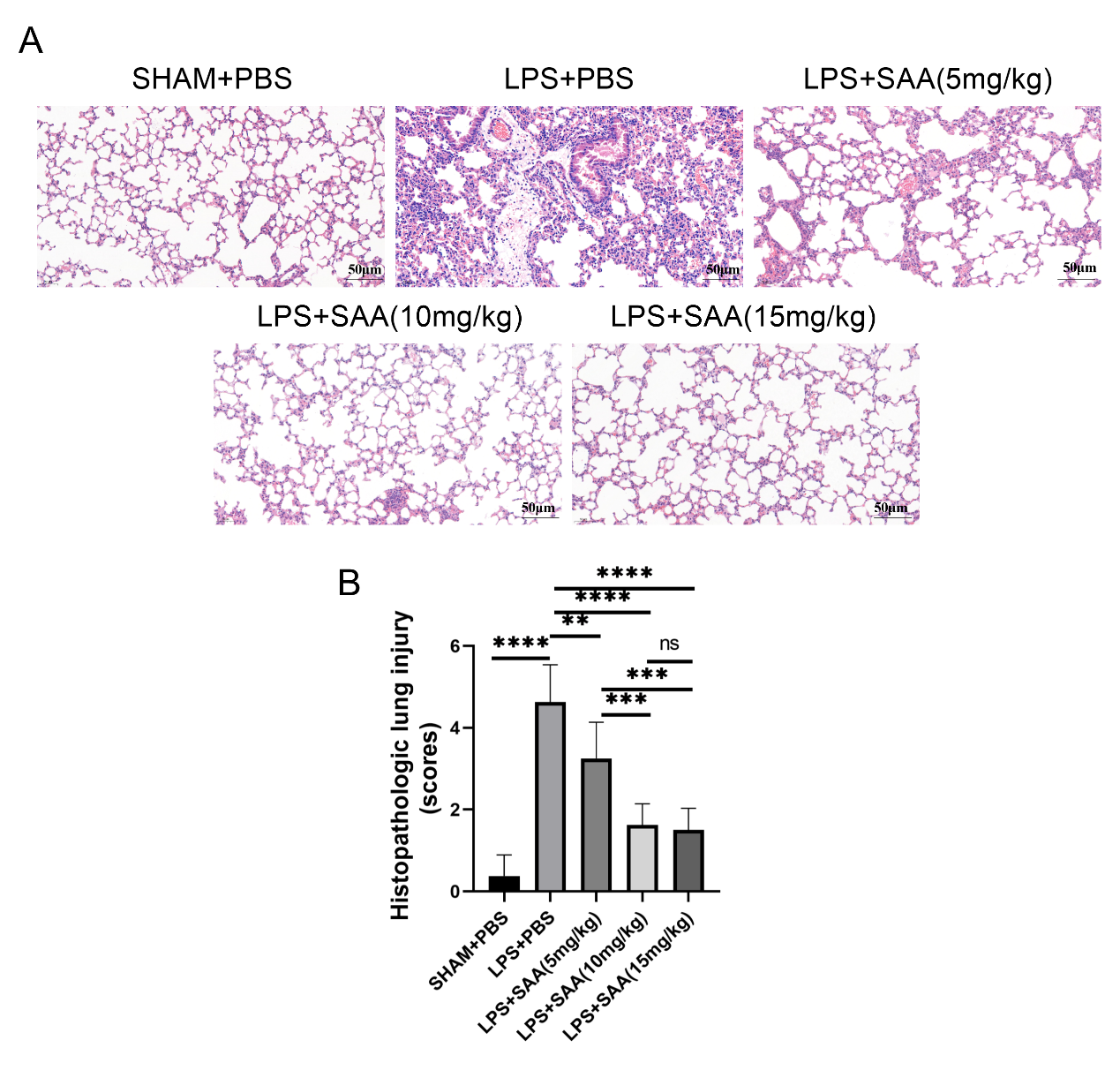


**Supplementary Fig 1. Effects of SAA at different doses on LPS-induced histopathologic changes in the lungs.** (A) Representative HE staining in histological sections of the lung at 24h after ALI modeling established by LPS (10mg/kg) and administration of different dose of SAA (5/10/15mg/kg) (Scale bar, 50μm). (B) The semi-quantitative scores of the histopathologic changes. **p < 0.01, ***p < 0.001, ****p < 0.0001 compared with indicated groups by ANOVA comparison. Data were presented as the mean ± SD (n = 6, each group).

**Table S1**

**Table S1. Information of patients with acute respiratory distress syndrome**

|  | Age (years) | Gender | Cause | PaO_2_/FiO_2_ [mmHg] |
| --- | --- | --- | --- | --- |
| ARDS 1 | 48 | Male | Pulmonary infection | 141 |
| ARDS 2 | 53 | Male | Pulmonary infection | 168 |
| ARDS 3 | 49 | Female | Pulmonary infection | 158 |
| ARDS 4 | 53 | Male | Pulmonary infection | 149 |
| ARDS 5 | 57 | Female | Pulmonary infection | 162 |
